# Quantifying Remission Probability in Type 2 Diabetes Mellitus

**DOI:** 10.3390/clinpract11040100

**Published:** 2021-11-09

**Authors:** Sanjay Kalra, Ganapathi Bantwal, Nitin Kapoor, Rakesh Sahay, Saptarshi Bhattacharya, Beatrice Anne, Raju A Gopal, Sunil Kota, Ashok Kumar, Ameya Joshi, Debmalya Sanyal, Mangesh Tiwaskar, Ashok Kumar Das

**Affiliations:** 1 Department of Endocrinology, Bharti Hospital, Karnal 132001, India; 2 Department of Endocrinology, St Johns Medical College & Hospital, Bengaluru 560034, India; mallyaganapathi@rediffmail.com; 3 Department of Endocrinology, Diabetes and Metabolism, Christian Medical College, Vellore 632004, India; nitin.endocrine@gmail.com; 4 Non Communicable Disease Unit, The Nossal Institute for Global Health, Melbourne School of Population and Global Health, University of Melbourne, Victoria 3010, Australia; 5 Department of Endocrinology, Osmania Medical College and Hospital, Hyderabad 500095, India; sahayrk@gmail.com; 6 Department of Endocrinology, Max Healthcare, New Delhi 110092, India; saptarshi515@gmail.com; 7 Department of Endocrinology, Nizams Institute of Medical Sciences, Hyderabad 500082, India; maglarne@yahoo.com; 8 Department of Endocrinology, Endodiab Clinic, Kozhikode 673016, India; drrajugopal@gmail.com; 9 Department of Endocrinology, Diabetes and Endocare Clinic, Berhampur 760004, India; diab.endocare@gmail.com; 10 Department of Endocrinology, CEDAR Diabetes Thyroid & Hormone Clinic Panipat, Panipat 132103, India; ashoka08@gmail.com; 11 Department of Endocrinology & Diabetes, Bhaktivedanta Hospital and Research Institute, Mumbai 401107, India; ameyaable@gmail.com; 12 Department of Endocrinology, KPC Medical College, Kolkata 700032, India; drdebmalyasanyal@gmail.com; 13 Department of Diabetology, Shilpa Medical Research Centre, Mumbai 400068, India; tiwaskar@gmail.com; 14 Department of Endocrinology & Medicine, Pondicherry Institute of Medical Sciences, Puducherry 605014, India; ashokdas82@gmail.com

**Keywords:** T2DM remission, reversal, type 2 diabetes mellitus, lifestyle interventions, bariatric surgery

## Abstract

Type 2 diabetes mellitus (T2DM) is a chronic progressive disorder and is associated with significant morbidity and mortality. The concept of T2DM remission and the reversal of diabetic parameters to normal levels has been gaining momentum over the past years. T2DM remission is increasingly being recognized by various global guidelines. Multiple models have been developed and validated for quantifying the extent of remission achieved. Based on favorable clinical evidence, T2DM remission can be considered as the therapeutic goal in diabetes management and, in select cases, as an alternative to expensive treatment options, which can be burdensome as T2DM progresses. This narrative review discusses the available strategies, such as lifestyle interventions, physical activity, bariatric surgery, medical nutrition therapy, and non-insulin glucose-lowering medications, for achieving T2DM remission. Although the concept of T2DM remission has emerged as a real-world option, effective implementation in routine clinical practice may not be feasible until long-term studies prove the efficacy of different approaches in this regard.

## 1. Diabetes: A Major Health Burden

Diabetes is increasingly being recognized as a major global health epidemic [1]. According to the International Diabetes Federation (IDF), about 463 million adults across the world were living with diabetes in 2019. The global prevalence of diabetes is expected to rise from 9.3% in 2019 to 10.9% in 2045. In 2019, there were an estimated 4.2 million deaths worldwide due to diabetes [2]. The complications associated with diabetes are accountable for significant morbidity and mortality. The chronic complications of diabetes include macrovascular complications, such as stroke, cardiovascular disease, and peripheral artery disease, and microvascular complications, such as nephropathy, neuropathy, and retinopathy [1]. Diabetes also imposes a significant economic burden, with an estimated health expenditure of USD 760.3 billion in 2019 [2]. People with diabetes are at a high risk for several cardiometabolic ailments and all-cause mortality [3]. Fortunately, a healthy lifestyle and the maintenance of a healthy body weight can prevent or delay diabetes [3]. 

## 2. Diabetes: Preventable, Curable, or Both?

In simple terms, prevention is defined as the aversion of development of a pathological state. It includes all measures, including definitive therapy to curtail the progression of the disease, at any stage of its course [4]. Since the 1980s, there has been growing interest in the prevention of diabetes through lifestyle changes [5]. This was re-emphasized in 2004 through the recommendations of the Diabetes and Nutrition Study Group (DNSG) of the European Association for the Study of Diabetes (EASD) [5,6]. In fact, several randomized controlled trials (RCTs) have shown that diabetes, specifically type 2 diabetes mellitus (T2DM), is preventable or that its onset can be significantly delayed by reducing body weight, increasing physical activity, and diet modification or with metformin use [5,7,8,9]. A systematic review of RCTs concluded that lifestyle modification can prevent T2DM, and the reduced risk is maintained for several years after the active intervention [5]. The Mediterranean dietary pattern or recommendation-based healthy dietary changes can be recommended for the long-term prevention of diabetes [5].

The term cure is defined as the restoration of health without any evidence of the disease process when evaluated using any of the available criteria to diagnose the condition or disease in question [10]. Unfortunately, a “cure” for diabetes is not perceived as being able to materialize in the near future. Therefore, the term “cure” is currently not applicable to T2DM, as it has been well documented that the condition has the potential for recurrence [11]. Therefore, it would be misleading to portray the “cure of diabetes” as a treatment goal for individuals with diabetes [12].

## 3. Reversal and Remission of T2DM: Are They Synonymous?

Although diabetes was considered as a chronic and irreversible condition in the past, there has been a shift in the paradigm of management goals in diabetes care. Given that diabetes is incurable, the best expected outcome is slowing the inevitable progression of diabetes or the amelioration of its symptoms [11]. The possibility of reversing T2DM was first indicated decades ago based on the outcomes noted in bariatric surgery patients. Pories et al. showed that in obese people with T2DM, blood glucose levels were normalized following bariatric surgery. About 90% of the study population remained free of diabetes after 10 years [13,14].

The reversal of diabetes is defined as the maintenance of glycated hemoglobin (HbA1c) levels under the diabetic threshold of 6.5% for a prolonged period of time without the use of any medications for glycemic control [11]. The global report on diabetes by the World Health Organization (WHO) has acknowledged diabetes reversal and the fact that it can be achieved through calorie restriction and weight loss [15]. Studies have shown that the reversal of diabetes can be achieved by using bariatric surgery or other approaches, such as carbohydrate restriction or low-calorie diets (LCD) [11]. 

In the available literature, “diabetes reversal” is used almost synonymously with “diabetes remission” [11]. The reversal of the parameters of diabetes, such as hyperglycemia, is termed as “diabetes remission” [12]. Although both the terms “remission” and “reversal” are commonly used in the field of diabetes care, recent consensus supports the use of “remission” in the context of T2DM [10,12]. 

## 4. T2DM Remission: Understanding the Basics

The remission of T2DM is suggested to be the primary clinical goal in the management of diabetes, and it should be promoted as an ideal therapeutic endpoint [12]. There is ample evidence to substantiate the feasibility of achievement of remission in T2DM. There are several means to accomplish remission in T2DM, all of which require the achievement of a sustainable weight loss [10].

### 4.1. Defining T2DM Remission

To understand the concept of T2DM remission, it is essential to have an appropriate and clear definition [12]. The first definition of T2DM remission was proposed by Buse et al. in 2009, stating that remission is the achievement of a glycemic level below the diabetic range, in the absence of any pharmacologic or surgical therapy [16]. A simpler definition was proposed by Nagi et al. in 2019 as the achievement of glycemia lower than the threshold value currently used for the diagnosis of T2DM, which is sustained for a minimum duration of six months after discontinuation of all glucose-lowering therapies [10]. Recently, a more contemporary definition has been proposed by Kalra et al., wherein T2DM remission has been defined as a healthy clinical state associated with the achievement of HbA1c below the targeted threshold, which is maintained for a minimum period of six months, with or without the continuous use of metformin and/or lifestyle changes, provided the condition is not due to comorbidities, concomitant therapies, or complications [12]. Recently, the consensus report of an international expert group convened by the American Diabetes Association has referred to remission as the sustained metabolic improvement in T2DM to nearly normal levels. Remission has been defined as the return of HbA1c levels to <6.5%, which occurs either spontaneously or after an intervention and which persists for ≥3 months in the absence of usual glucose-lowering therapy. In case HbA1c is considered an unreliable marker of glycemic control, fasting plasma glucose level <7.0 mmol/L (<126 mg/dL) or estimated HbA1c <6.5% as calculated from continuous glucose monitoring values can be used as an alternative criteria for defining remission [17]. 

### 4.2. Levels of T2DM Remission

T2DM remission can be broadly categorized as partial or complete. Previously, the achievement of glycemic levels below the threshold value for the diagnosis of diabetes, i.e., prediabetic glycemic level, was considered as partial remission. Complete remission implied the maintenance of normal glycemic parameters for a minimum duration of one year [12,16]. The term “prolonged remission” was used to define complete remission that was maintained over a duration of more than five years [16].

Recently, the categorization of diabetes remission has been based on metabolic, clinical biochemical, and pharmacologic parameters [12]:

- Metabolic remission: the reversal of insulin secretory defect or insulin resistance.
- Clinical or partial remission: the achievement of HbA1c below an individual’s set target.
- Biochemical or complete remission: the achievement of HbA1c below the threshold value for the diagnosis of diabetes.
- Pharmacological remission: Absence of the requirement of any drug therapy.

### 4.3. Predictors and Quantification of T2DM Remission

Although certain modalities can help in diabetes remission, all diabetes patients do not achieve desirable outcomes. An evaluation of the predictors of remission is hence critical for optimal outcomes. For example, factors that influence diabetes remission following bariatric surgery include higher baseline BMI, younger age, shorter diabetes duration, and better glycemic control before surgery [18]. However, traditionally, only patients with BMI ≥35 Kg/m^2^ with uncontrolled diabetes were considered as potential candidates for bariatric surgery [19]. The DiRECT trial identified some predictors of T2DM remission in the intervention arm, amongst which early and greater weight loss and being on fewer antidiabetic medications were the strongest predictors. While individuals with lower HbA1c and men were more successful at attaining remission, individuals with anxiety and/or depression had lower success [20]. Again, in patients on intensive insulin therapy, the key predictor of achieving sustained drug-free remission was early intervention, particularly within the first two years of diagnosis [21].

Initially, different sets of predictive factors, such as younger age, shorter duration of diabetes, higher baseline body mass index (BMI), and better glycemic control, were used to determine the rate of diabetes remission. Later, three different models were introduced for the prediction of diabetes remission, which were more practical for clinical use [18]. The pre-operative diabetes remission (DiaRem) score was developed for predicting remission rate in persons with T2DM who had undergone Roux-en-Y gastric bypass (RYGB) surgery [22]. This scoring system is based on parameters such as HbA1c, insulin usage, age, and medications [23]. The scores range from 0 to 22 and are divided into five groups based on the probability of remission. The probability of partial and complete remission of T2DM based on the DiaRem scores are defined as follows: 87%, 66%, 32%, 16%, and 5% for scores 0–2, 3–7, 8–12, 13–17, and 18–22, respectively [18].

The diabetes surgery score or the ABCD score was proposed by Lee et al., wherein A stands for age, B stands for BMI, C stands for C-peptide level, and D stands for duration of diabetes [23,24]. For the duration of diabetes, C-peptide levels, and for BMI, a four-point score ranging from 0 to 3, are used. While for age, a one-point score is used [23]. After adding the points for all variables, the final ABCD score ranges from 0 to 10 points, with higher scores predicting a higher probability of remission following gastric bypass surgery [23].

The Individualized Metabolic Surgery (IMS) score was devised to guide the selection of procedure for individuals with T2DM based on long-term glucose control (>5 years) [18,25]. The nomogram for score calculation is based on the four predictors of long-term remission, i.e., the number of diabetes medications, duration of diabetes, insulin use, and glycemic control (HbA1c <7%). Scores of ≤25, 25–95, and >95 indicate mild, moderate, and severe disease, respectively [18,25].

## 5. Strategies to Achieve T2DM Remission

Based on the available evidence, the remission of T2DM can be achieved through lifestyle modifications, physical activity, and bariatric surgery [11,26,27]. The 2014 recommendations of the US Preventive Services Task Force support the need for nutritional counseling and physical activity for adults at risk of cardiovascular diseases (CVDs) [28]. The American Heart Association (AHA) suggests the implementation of behavior change techniques, such as goal setting and self-monitoring for promoting lifestyle change [3,29]. The ADA suggests referring high-risk individuals with diabetes to health programs for increasing physical activity and to target weight loss [3,30].

### 5.1. Lifestyle Modifications

The existing evidence indicates that the remission of T2DM can be achieved through sufficiently intensive lifestyle modifications [27]. The lifestyle modifications explored for diabetes remission are a low-carbohydrate diet and low-calorie diet (LCD) and medical nutrition therapy (MNT) [11].

- Low-Calorie Diet

Studies have substantiated the effectiveness of calorie-restricted diets in the successful achievement of T2DM remission, even in the absence of any pharmacological intervention [31]. Calorie restriction facilitates the normalization of hepatic glucose output; beta-cell function, including secretory capacity; visceral fat; indices of insulin resistance; and insulin sensitivity [31]. Findings from a clinical audit of 12 obese persons with T2DM (baseline median HbA1c 9%) receiving 12 weeks of LCD showed that 50% of the study population achieved HbA1c within the non-diabetes range (<6.5%) at the end of 12 weeks on LCD, without using any antidiabetic medications (*p* < 0.001) [31].

In a study involving 12 overweight/obese individuals with recently diagnosed (<1 year) T2DM, the effects of a six-month intensive lifestyle program comprising both physical exercise and calorie restriction were assessed. At least partial remission was achieved by 67% of the total study population and 80% of the participants who completed the study. One participant achieved complete remission with an HbA1c level of 5.6% [32].

Another study evaluated the effects of restricted diet at 1, 4, and 8 weeks on the reversal of insulin resistance and beta-cell failure in 11 individuals with T2DM. The normalization of both hepatic insulin sensitivity and beta-cell function was achieved. The study concluded that the metabolic derangements underlying T2DM could be potentially reversed by acute dietary restriction on energy intake [33]. In general, individuals with lower baseline HbA1c, taking lesser hypoglycemic medications, and with a shorter duration of diabetes are more frequently reported to have achieved diabetes remission [11].

- Medical Nutrition Therapy

Owing to the direct correlation between diabetes management and diet, MNT advised by a registered dietician can potentially complement the need for traditional anti-diabetic medications [26]. Medical nutrition therapy is defined as “nutritional diagnostic, therapy, and counselling services for the purpose of disease management, which are furnished by a registered dietician or nutrition professional.” It is a supportive process that helps establish goals, set priorities, and create individualized action plans that promote self-care in individuals with diabetes [26]. The components of MNT are nutrition screening/referral, assessment, diagnosis, intervention, monitoring, evaluation, documentation, and outcome management systems. It is important to note that MNT is not the same as diabetes self-management training and also may require ethnicity-specific adjustments. [26,34]. The effectiveness of MNT in T2DM was demonstrated by the Diabetes Remission Clinical Trial (DiRECT), which showed that weight loss associated with lifestyle intervention caused diabetes remission in 46% of study participants after one year. The remission rate was linked to the extent of weight loss, increasing progressively from 7% to 86% after 1 year, concurrent with an increase in weight loss from 0–5 Kg, 5–10 Kg, 10–15 Kg, and ≥15 Kg [35].

- Physical Activity

It has been substantiated by ample evidence that short-duration T2DM can be reversed to non-diabetic blood glucose levels through significant weight loss. Further, sustainable daily physical activity is an essential component of long-term weight control [36]. The Nutrition Practice Guideline (NPG) recommends an individualized physical activity plan for diabetes. The guideline suggests the achievement of ≥150 min/week of moderate-intensity (50–70% of maximum heart rate) aerobic physical activity for at least 3 days per week, without any gap of two consecutive days without exercise [37]. It is also important to advise the maintenance of usual physical activity and to avoid any sudden exercise program during the weight-loss phase [36].

It is not only important to design and recommend strict lifestyle measures for the reversal of diabetes but equally important to ensure that these interventions are cost effective, less resource intensive, and largely scalable at the population level. A recently conducted randomized controlled cohort study in southern India assessed such a peer-led, low-cost, scalable, lifestyle intervention in individuals of South Asian descent and found it to be helpful in improving diabetes and lowering other cardiovascular risk factors [38,39,40].

### 5.2. Bariatric Surgery

Bariatric surgery is well recognized as a potential treatment option for morbid obesity and associated metabolic disorders, such as T2DM. Bariatric surgery for the management of adults with T2DM is approved for those with BMI >40 or >35 kg/m^2^ along with obesity-related comorbidities. The most common bariatric surgeries are sleeve gastrectomy (SG) and RYGB [11]. The five-year follow-up outcomes of the Sleeve vs. Bypass (SLEEVEPASS) randomized clinical equivalence trial reported partial or complete remission of T2DM in 45% of RYGB patients and 37% of SG patients [41]. These findings were aligned with other similar studies that demonstrated long-term T2DM remission in about one-third of patients undergoing bariatric surgery [11]. The large prospective cohort study, Longitudinal Assessment of Bariatric Surgery-2 (LABS-2), evaluated the incidence and remission rates of diabetes following laparoscopic gastric banding (LAGB) and RYGBP. After 3 years, 30.2% of LAGB patients and 68.7% of RYGB patients were in remission, and the remission was progressively higher with an increase in weight loss post-surgery [42].

## 6. Role of Medications in Achieving T2DM Remission

Pharmacotherapy is an essential component of non-surgical modalities for T2DM remission. There are three classes of non-insulin glucose-lowering drugs: insulin sensitizers, nutrient load reducers, and insulin secretagogues [43]. Therapeutic agents that lower insulin resistance in people with T2DM are known as insulin sensitizers [44]. Insulin sensitizers can be direct acting, such as metformin and pioglitazone, or indirect acting through counter-regulatory hormone pathways, such as pramlintide and bromocriptine. Similarly, insulin secretagogues are either direct acting, such as sulfonylureas and meglitinides, or indirect acting via the incretin pathway, such as dipeptidyl peptidase-4 inhibitor (DPP4i) and glucagon-like peptide 1 receptor agonists (GLP1RAs). Nutrient load reducers are also of two types: absorption enhancers, such as alpha glucosidase inhibitors, and excretion enhancers, such as sodium–glucose cotransporter 2 inhibitors (SGLT2is) [43]. 

Another concept of calorie restriction in patients with T2DM involves the use of calorie restriction mimetics [45,46]. Calorie restriction (CR) is defined as a decreased amount of calorie consumption compared to previous intake or that which is comparable to matched controls, without any malnutrition [46]. A calorie restriction of 30–40% improved morbidity and mortality, particularly by reducing the development of glucose intolerance and cardiovascular disease [46]. Calorie restriction mimetics (CRMs) represents a class of medication that induces similar effects to those of CR, including the de-acceleration of aging and the improvement of health and lifespan [46]. In this regard, several non-insulin glucose-lowering drugs can also be considered as CRMs, such as alpha glucosidase inhibitors, which delay the uptake of glucose from the gut by delaying the breakdown of complex carbohydrates, appetite-suppressing GLP1RA, and calorie-wasting SGLT2i [45,46].

### 6.1. Insulin Sensitizers in T2DM Remission

A study assessed the effects of metformin and pioglitazone combination on achieving long-term glycemic control in 373 people with T2DM (<24 months duration). The participants were administered a triple-drug combination of metformin, gliclazide, and pioglitazone. Once controlled, the doses of gliclazide were reduced if the blood glucose levels decreased. Pharmacological remission was defined as the maintenance of blood glucose levels in the normal range for more than six months, without the use of sulfonylureas. After using triple-drug therapy, 36.3% of the study population achieved pharmacological remission. The study inferred that insulin sensitizers, such as pioglitazone in combination with metformin, can induce diabetes remission in individuals with T2DM [47].

### 6.2. Sodium–Glucose Cotransporter-2 Inhibitors in T2DM Remission

In a case report, a 43-year-old man with a BMI of 26 kg/m^2^, advanced insulin resistance, HbA1c 10.3%, fatty liver, and pancreatic beta-cell dysfunction was given comprehensive therapy, including metformin and SGLT2i, for 18 months. After 18 months of comprehensive therapy, the person’s glycemic control was nearly normal (HbA1c 5.3%), and their BMI was lowered to 21.3 kg/m^2^. These effects were observed in spite of the discontinuation of all glucose-lowering medications. This real-world clinical evidence demonstrated that diabetes remission and normal body weight could be effectively achieved in persons with newly diagnosed T2DM by a combination therapy of metformin and SGLT2i [48].

### 6.3. The Alpha Glucosidase Inhibitor: Another Potential Candidate for T2DM Remission

In an RCT involving 187 drug-naïve persons with newly diagnosed T2DM, the efficacy and safety of metformin monotherapy was compared with a fixed-dose combination of voglibose plus metformin. At 24 weeks, the reductions in HbA1c levels were −1.62% ± 0.07% in the voglibose plus metformin group and −1.31% ± 0.07% in the metformin group (*p* = 0.003). Weight loss was also significantly higher in the voglibose plus metformin group as compared to metformin monotherapy (−1.63 kg vs. −0.86 kg, *p* = 0.039). Further, the number of people achieving target HbA1c levels was significantly higher in the voglibose plus metformin group (*p* = 0.002 for HbA1c <6.5% and *p* = 0.039 for HbA1c <7%). The study showed that voglibose could be useful in achieving T2DM remission [49].

An observational study evaluated the effectiveness of voglibose in preventing the development of T2DM in high-risk individuals. A total of 66 eligible persons with impaired glucose tolerance, also following regular exercise and a standard diet, were randomly assigned to receive voglibose or placebo. With an average treatment of 48.3 weeks, voglibose was better than placebo (*p* = 0.0024), as voglibose-treated persons had a lower risk of progression to T2DM, and a greater number of persons achieved normoglycemia in this group as compared to the placebo group. The study showed that lifestyle modification plus voglibose can lower the risk of T2DM development in high-risk individuals with impaired glucose tolerance [50].

## 7. T2DM Remission as the Key Treatment Goal: The Future Is Not So Far

The paradigm of diabetes care and treatment has been evolving continuously. The approaches for treating diabetes, measuring glycemia, and assessing glycemic targets have been changing, as evident from the introduction of practices and concepts such as bariatric surgery, continuous glucose monitoring system, and the time range of diabetes care [12]. Similarly, there has been a constant effort to assess the potential of achieving diabetes remission through different modalities in real-world practice. With gradual accumulation of clinical evidence, hopes regarding the achievement of diabetes remission have been raised [12]. According to recent studies, comprehensive lifestyle changes, specific diets, weight reduction, and short-term intensive glucose-lowering therapy were associated with the achievement of diabetes remission in up to 50% of participants [51]. Therefore, achieving diabetes remission is possible in real-world practice and can be considered as an alternative to traditional diabetes management. However, further clinical studies and an in-depth understanding of the mechanisms underlying diabetes remission with different strategies are warranted. Healthcare providers are also required to be made aware and educated about the possibility of diabetes remission in their practice [11].

## 8. Patient Selection Criteria for Different Remission Strategies

As all diabetes patients do not equally benefit from different remission modalities, patients should be carefully selected for each remission strategy. According to the recent guidelines by the ADA, metabolic surgery should be a recommended for screened surgical T2DM patients with BMI ≥40 Kg/m^2^ (BMI ≥37.5kg/m^2^ in Asian Americans) and in adults with BMI 35.0–39.9 kg/m^2^ (32.5–37.4 kg/m^2^ in Asian Americans) who failed to achieve durable weight loss and improvement in comorbidities (including hyperglycemia) with non-surgical methods [52]. Patients selected for metabolic surgery should be assessed for social and situational circumstances and comorbid psychological conditions, which can potentially interfere with the outcomes of metabolic surgery [52].

Regarding meal patterns, every diabetes patient should be provided with individualized meal planning. The ADA suggests that both at the time of diagnosis and as needed throughout the lifespan, every individual with diabetes should be referred to a registered dietician nutritionist for individualized MNT [53]. Patient selection should be based on the evidence of the benefits from a specific remission strategy [54]. Patient selection criteria for a low-carbohydrate diet could be prediabetes patients (HbA1c 5.7–6.5%) or T2DM patients (HbA1c >6.5%) [54]. Physical activity in addition to exercise should be prescribed and recommended for every individual with diabetes. The recommendations should be tailored as per the specific needs of every individual. Factors such as the presence of diabetes-associated health complications, age, type of diabetes, and activity levels should be considered for specific recommendations related to physical activity [53].

Non-insulin glucose-lowering drugs have a CRM effect, which is helpful in individuals with obesity or “maladaptive anabolism” or metabolic syndrome. However, they could be deleterious in persons with minimal insulin reserves and malnutrition or in those who are calorie-deprived or cachexic. It would be inappropriate to add SGLT2i to an energy-malnourished carbohydrate-deprived person. Similarly, the use of AGIs will not be useful in individuals on a low-fat diet. These drugs may be strongly favored for persons with insulin resistance and those who are unable to exercise or control their appetite [46].

## 9. Conclusions

Diabetes mellitus poses a significant global health and economic burden. Although incurable, options such as diabetes prevention, delay in onset and progression, and remission or reversal have been widely embraced in the field of diabetes care. The concept of T2DM remission has been soaring high for more than a decade, as evident from the increasing number of clinical studies. Although the concept of remission is not well defined, efforts have been made to aptly define and quantify the achievement of remission in persons with T2DM. Lifestyle modifications, such as diet modifications and physical activity modifications, along with bariatric surgery, are pivotal components for achieving T2DM remission. Newly diagnosed people with T2DM should receive formal nutritional counseling and lifestyle guidance to accomplish weight loss and lifestyle improvement. Glucose-lowering agents, such as AGI, SGLT2i, pioglitazone, and metformin, showed their potential in achieving T2DM remission. In summary, achieving diabetes remission is no more a myth but a reality. However, long-term studies on the effectiveness of various remission strategies are required. 

## Data Availability

Data sharing is not applicable to this article as no datasets were generated or analyzed during the current study.
